# Supplementary material for: Genomic diversity across the Rickettsia and ‘Candidatus Megaira’ genera and proposal of genus status for the Torix group
Source: Nat Commun. 2022 May 12;13:2630. doi: 10.1038/s41467-022-30385-6 (PMC9098888; doi:10.1038/s41467-022-30385-6)
Supplement: Supplementary file 1 — Supplementary Information [file 41467_2022_30385_MOESM1_ESM.pdf]

Supplementary Information for "Genomic diversity across the Rickettsia and 'Candidatus Megaira' genera and proposal of genus status for the Torix group."

Helen R Davison[1], Jack Pilgrim[1], Nicky Wybouw[2], Joseph Parker[3], Stacy Pirro[4], Simon Hunter-Barnett[1], Paul M Campbell, [1,5], Frances Blow [1,6], Alistair C Darby[1], Gregory D D Hurst[1] and Stefanos Siozios[1]*

Affiliations

1. Institute of Infection, Veterinary and Ecological sciences, University of Liverpool, Liverpool, L69 7ZB, United Kingdom

2. Terrestrial Ecology Unit, Department of Biology, Faculty of Sciences, Ghent University, Ghent, Belgium

3. Division of Biology and Biological Engineering, California Institute of Technology, 1200 E California Boulevard, Pasadena, CA 91125, USA

4. Iridian Genomes, Bethesda, MD, USA

5. School of Health and Life Sciences, Faculty of Biology Medicine and Health, the University of Manchester, Manchester, United Kingdom

6. Center for Genomics and Systems Biology, Department of Biology, New York University, New York, New York, USA

* Corresponding author (siozioss@liverpool.ac.uk)

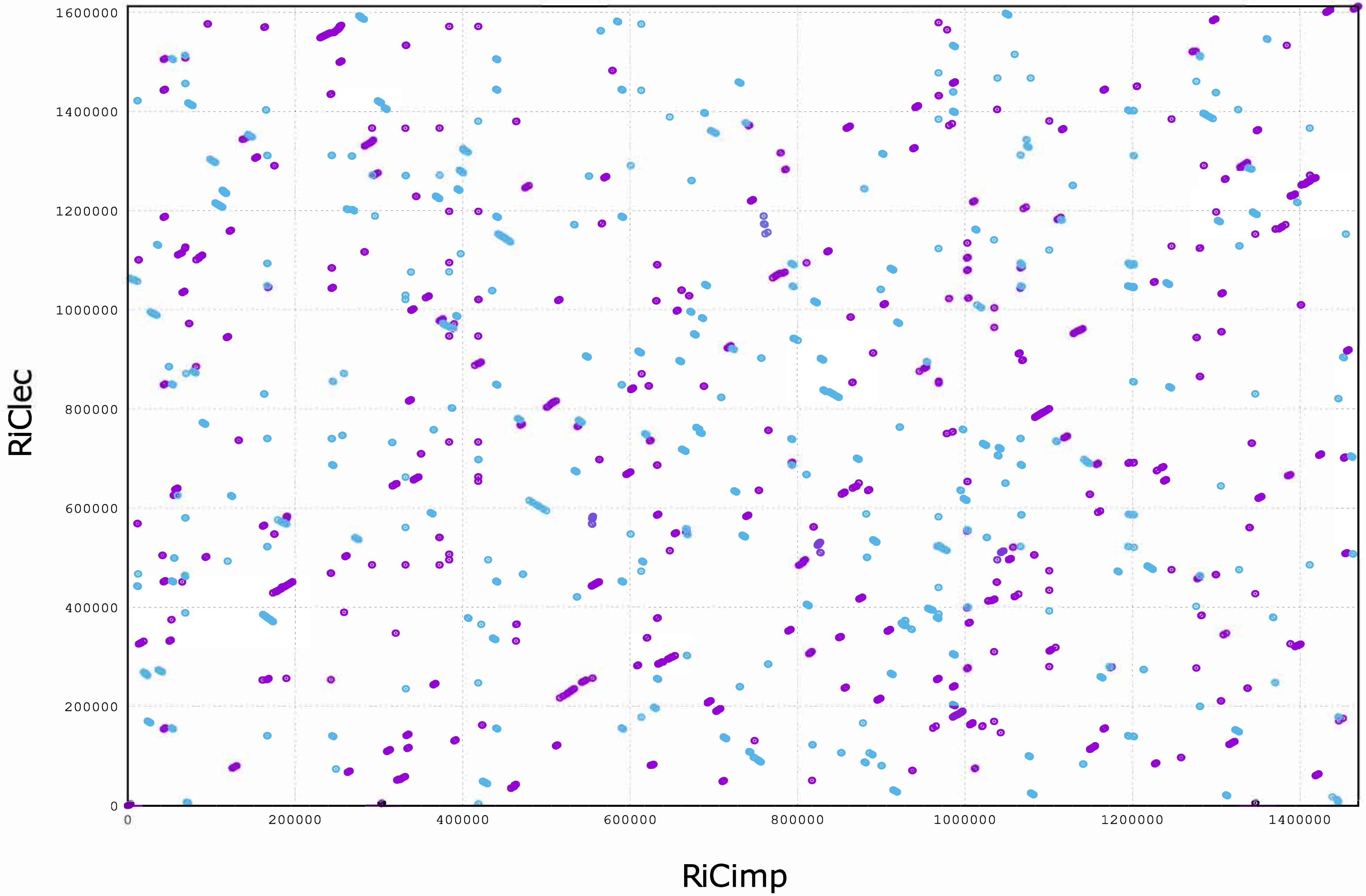

Supplementary Figure 1. Whole genome alignment between the complete Torix limoniae (RiCimp) and Torix Leech (RiClec) genomes reveals lack of synteny. Magenta represents forward matches and blue reverse matches https://doi.org/10.6084/m9.figshare.14866263.

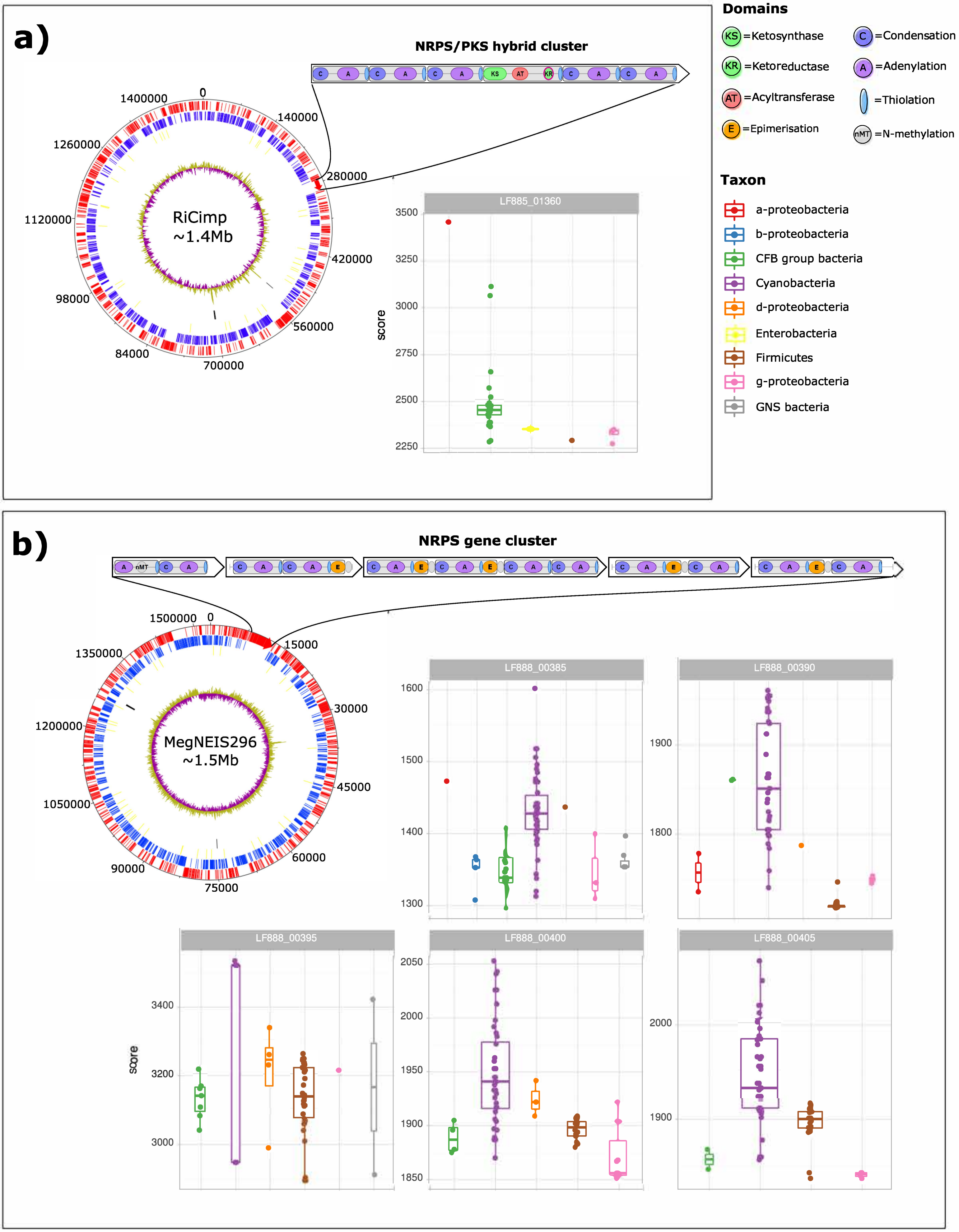

Supplementary Figure 2. The circular chromosomes of a) a Torix group Rickettsia (RiCimp) and b) a Ca. Megaira sp. (MegNEIS296). From outside to in, the circles represent: forward CDSs (Red), Reverse CDSs (blue), tRNAs (yellow) rRNAs (black), and GC content (green and magenta). Highlighted are the predicted domains that form non-ribosomal peptide synthase (NRPS) and hybrid non-ribosomal peptide synthase/ polyketide synthase (NRPS/PKS) gene modules. Modules define individual amino acids in the synthesised peptide and show the catalytic domains within modules. The plots beneath the predicted domains show the taxonomic distribution of the best blast hits for each predicted protein in the two NRPS systems (LF885_01360: n=49; LF888_00385: n=86, LF888_00390: n=69; LF888_00395: n=49, LF888_00400: n=80; LF888_00405: n=71). Center line, median; box limits, 25th and 75th percentiles; whiskers, ±1.5x interquartile range; points, data points. For the full resolution image: https://doi.org/10.6084/m9.figshare.14865570.

Ribosomal Proteins

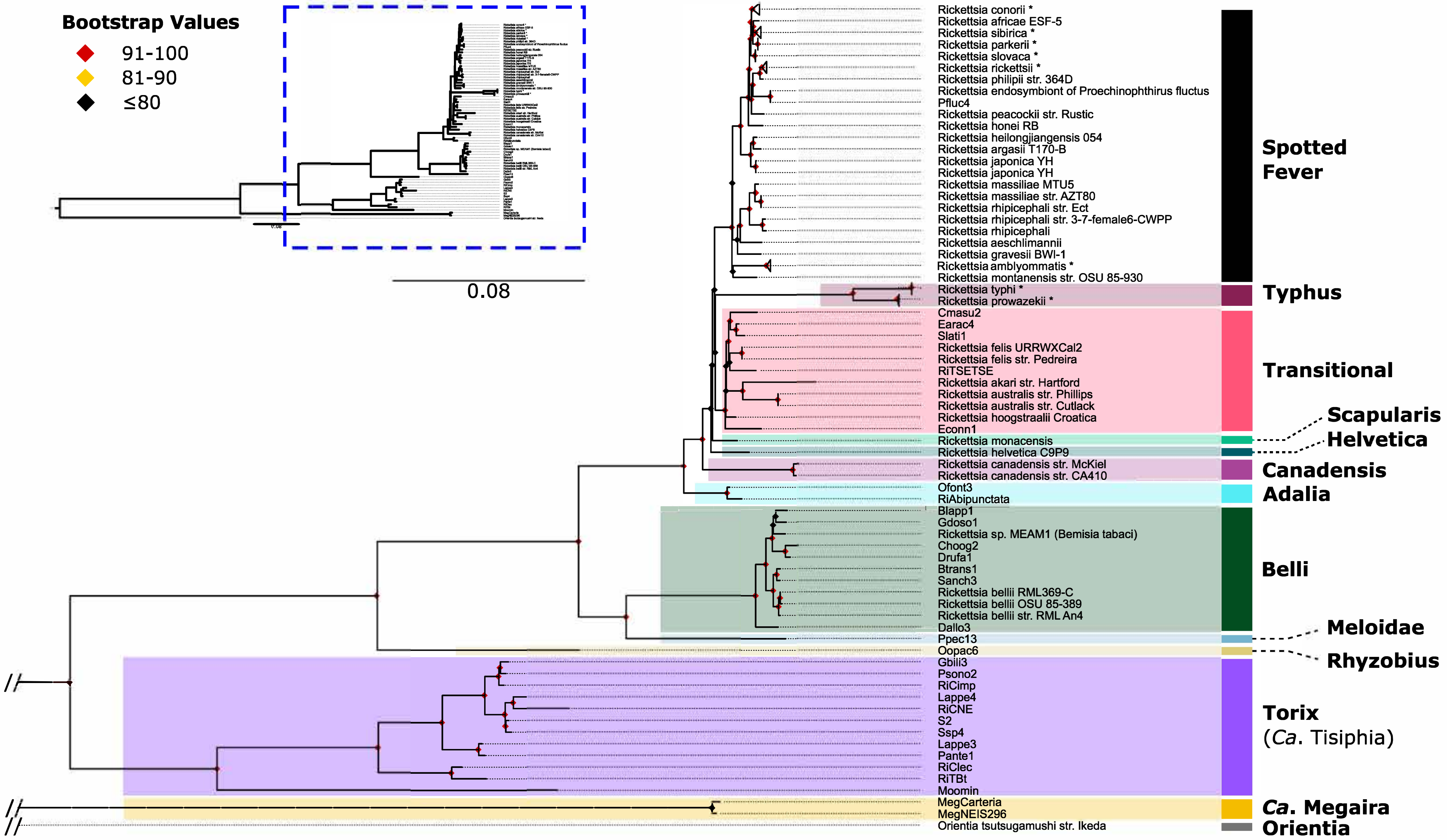

Supplementary Figure 3. Maximum likelihood (ML) phylogeny of *Rickettsia* and *Ca.* Megaira constructed from 43 ribosomal protein gene clusters extracted from the pangenome. New genomes are written in bold and bootstrap values based on 1000 replicates are indicated with coloured diamonds. Asterisks pangenome. New genomes are written in bold and bootstrap values based on 1000 replicates are indicated with coloured diamonds. Asterisks indicate collapsed monophyletic branches and "//" represent breaks in the branch. New complete genomes are: RiCimp, RiClec and MegNEIS296. https://doi.org/10.6084/m9.figshare.14865606

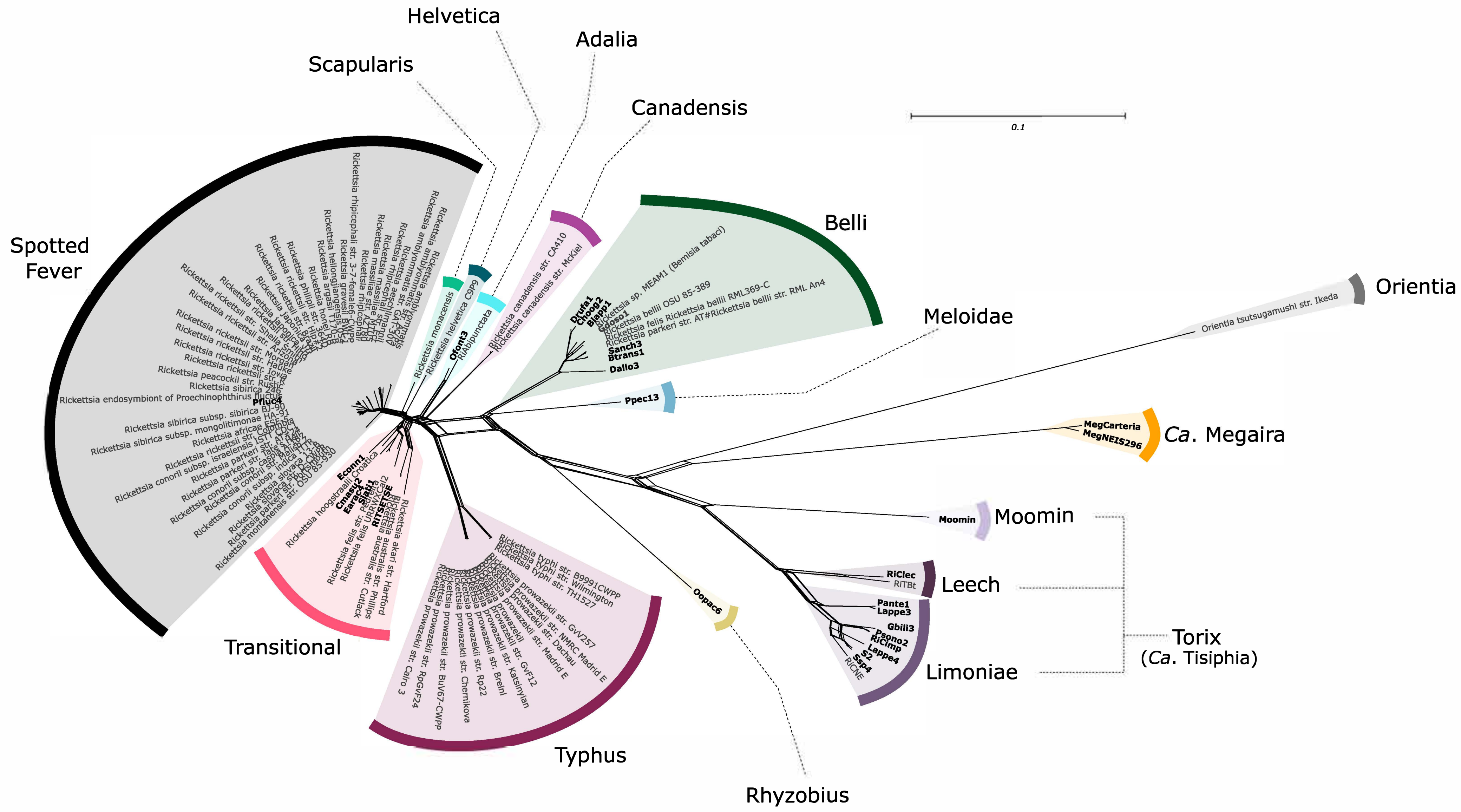

Supplementary Figure 4. Nearest Neighbour Network, displaying the distances between the 74 core gene sets across all 104 *Rickettsia, Ca.* Megaira genomes, and the outgroup Orientia. New genomes are indicated with bold text. A full resolution version can be found here: https://doi.org/10.6084/m9.figshare.15081975.

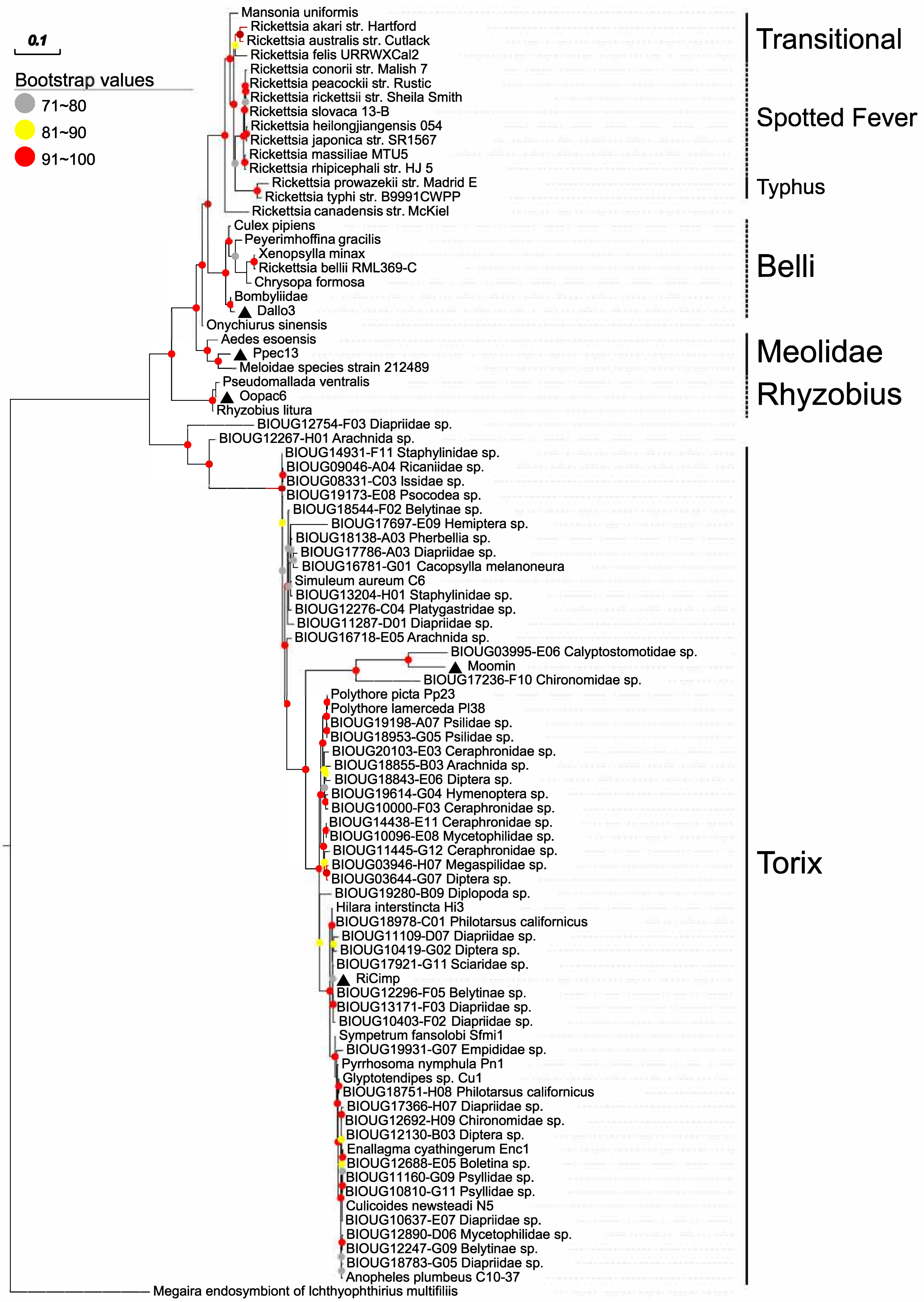

Supplementary Figure 5. Phylogram of a maximum likelihood (ML) tree of 90 *Rickettsia* multilocus profiles. The tree is based on 4 loci, 16S rRNA, 17Kda, gltA, and COI,under a partition model (2,781 bp total). https://doi.org/10.6084/m9.figshare.14865600

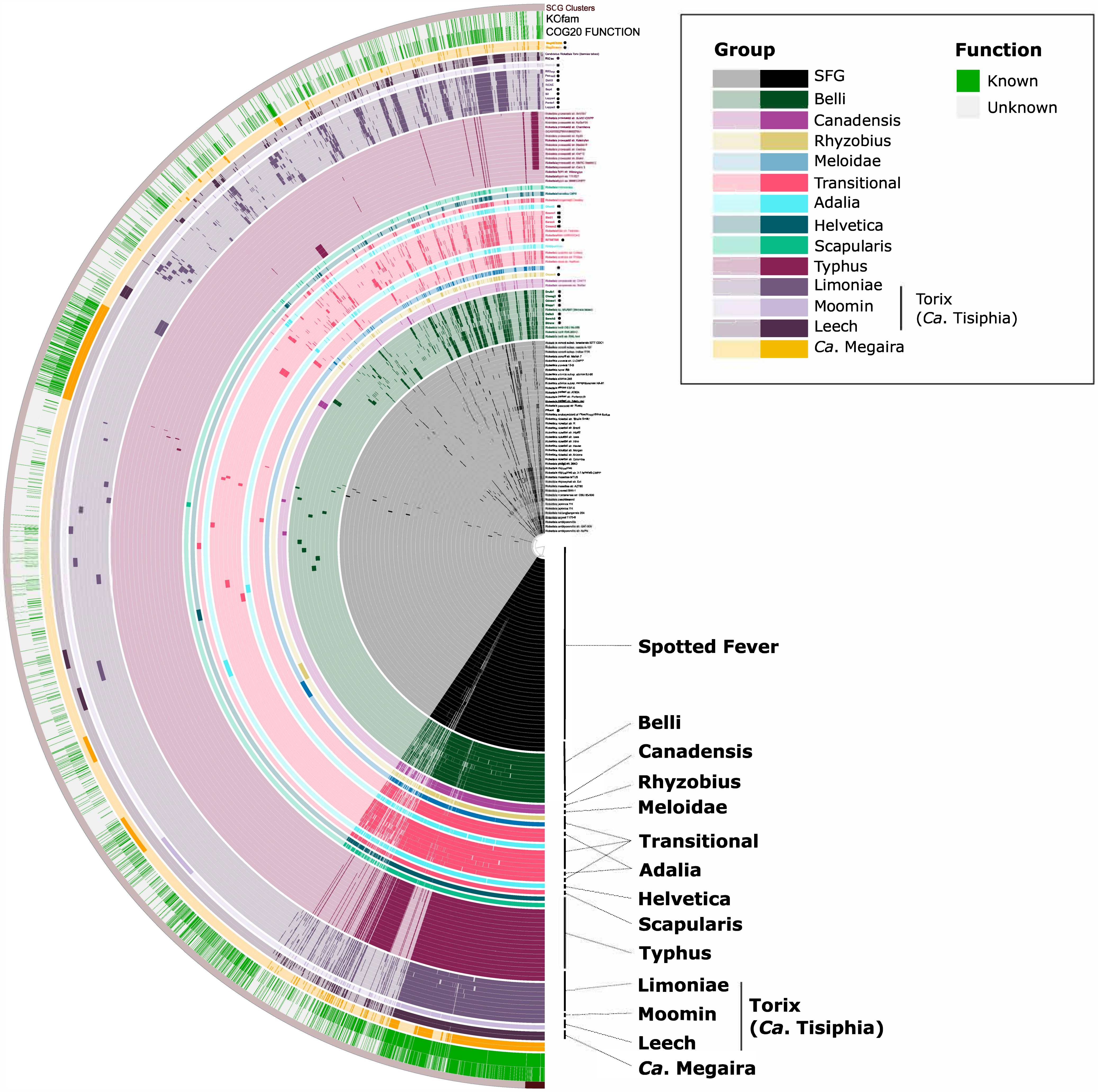

Supplementary Figure 6. Pangenome of 103 genomes including *Rickettsia,* Torix, and *Ca.* Megaira. New genomes are indicated by•· Each genome displays gene cluster presence/absence and is organised by gene cluster frequency. Group identity was assigned from phylogeny. SFG is Spotted Fever Gro0up. A full resolution version can be found here: https://doi.org/10.6084/m9.figshare.15081975.

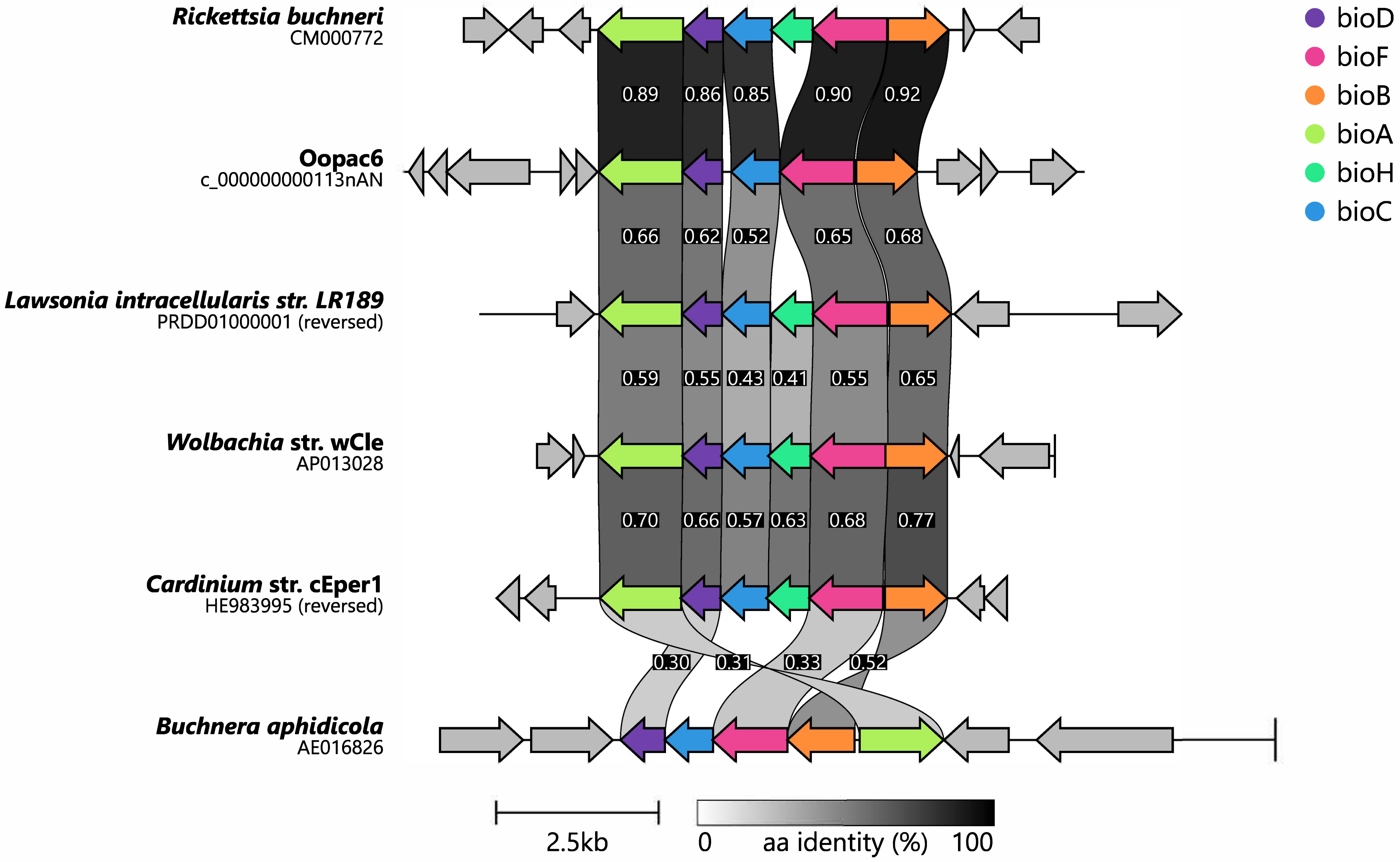

Supplementary Figure 7. Biotin operon of the Oopac6 Rhyzobius Rickettsia and its surrounding genes compared with other known biotin pathways in other related symbionts. Similarity scores in the black boxes refer to the percentage identity between the protein translations of the genes of the biotin operons, further illustrated by a greyscale bar. Optimal ordering of the operons was performed by hierarchical clustering of an all-vs-all similarity matrix, showing the closest relationships between all 6. https://doi.org/10.6084/m9.figshare.14865567
