# Supplementary material for: Genomic diversity across the Rickettsia and ‘Candidatus Megaira’ genera and proposal of genus status for the Torix group
Source: Nat Commun. 2022 May 12;13:2630. doi: 10.1038/s41467-022-30385-6 (PMC9098888; doi:10.1038/s41467-022-30385-6)
Supplement: Supplementary file 2 — Reporting Summary [file 41467_2022_30385_MOESM2_ESM.pdf]

nature portfolio

Corresponding author(s): Stefanos Siozios

Last updated by author(s): Apr 12, 2022

Reporting Summary

Nature Portfolio wishes to improve the reproducibility of the work that we publish. This form provides structure for consistency and transparency in reporting. For further information on Nature Portfolio policies, see our Editorial Policies and the Editorial Policy Checklist.

Statistics

For all statistical analyses, confirm that the following items are present in the figure legend, table legend, main text, or Methods section.

n/a	Confirmed	
☐	☒	The exact sample size (*n*) for each experimental group/condition, given as a discrete number and unit of measurement
☒	☐	A statement on whether measurements were taken from distinct samples or whether the same sample was measured repeatedly
☒	☐	The statistical test(s) used AND whether they are one- or two-sided<br>*Only common tests should be described solely by name; describe more complex techniques in the Methods section.*
☒	☐	A description of all covariates tested
☒	☐	A description of any assumptions or corrections, such as tests of normality and adjustment for multiple comparisons
☐	☒	A full description of the statistical parameters including central tendency (e.g. means) or other basic estimates (e.g. regression coefficient) AND variation (e.g. standard deviation) or associated estimates of uncertainty (e.g. confidence intervals)
☒	☐	For null hypothesis testing, the test statistic (e.g. *F*, *t*, *r*) with confidence intervals, effect sizes, degrees of freedom and *P* value noted<br>*Give P values as exact values whenever suitable.*
☒	☐	For Bayesian analysis, information on the choice of priors and Markov chain Monte Carlo settings
☒	☐	For hierarchical and complex designs, identification of the appropriate level for tests and full reporting of outcomes
☒	☐	Estimates of effect sizes (e.g. Cohen's *d*, Pearson's *r*), indicating how they were calculated

Our web collection on statistics for biologists contains articles on many of the points above.

Software and code

Policy information about availability of computer code

Data collection	SRA deposits (fastq files) were downloaded from the European Nucleotide Archive database using a custom perl script written by Michael Gerth and can be found here (https://github.com/gerthmicha/perlscripts/blob/master/sra_download.pl).
Data analysis	Guppy v4.0.15, NanoFilt v2.7.1, Flye v2.8.1, SOAPnuke version 2.1.4, megahit v1.2.9, MetaBAT2 v2.12.1, Pilon v1.23, CheckM v1.0.13, Anvi'o v7 (including pyANI and KEGG), samtools v1.11 IQ-TREE v2.1.2, R 3.4.4, AntiSMASH version 6.0, Blast+ v2.9.0, PhiPack v1.1, GTDB-Tk v.0.1.4, ComplexUpset v1.3.1, seqkit v2.0.0, CASAVA v1.8, Cutadapt v1.2.1, Sickle v1.200, DISCOVAR v50693, Bowtie2 v2.2, SPAdes v3.7.1, Blobology package v1.1 (no longer maintained last version is here: https://github.com/blaxterlab/blobology), Qualimap v2.2. Custom rapper scripts and R scripts listed in the supplementary data are available in github repositories (https://github.com/VibrantStarling/Code-used-to-extract-bacterial-genomes-from-invertebrate-genomes) and (https://github.com/SioStef/panplots).

For manuscripts utilizing custom algorithms or software that are central to the research but not yet described in published literature, software must be made available to editors and reviewers. We strongly encourage code deposition in a community repository (e.g. GitHub). See the Nature Portfolio guidelines for submitting code & software for further information.

Data

Policy information about availability of data

All manuscripts must include a data availability statement. This statement should provide the following information, where applicable:

- Accession codes, unique identifiers, or web links for publicly available datasets
- A description of any restrictions on data availability
- For clinical datasets or third party data, please ensure that the statement adheres to our policy

All assembled genomes have been deposited in GenBank. The original genomes and raw reads produced in this study can be accessed at Bioproject accession

March 2021

PRJNA763820. Genome assemblies produced from previously published third party data can be accessed at Bioproject PRJNA767332. Supplementary data and full resolution figures can be accessed on figshare here: https://doi.org/10.6084/m9.figshare.c.5518182.v1. All accessions used are listed in the supplementary data and in table 2.

Field-specific reporting

Please select the one below that is the best fit for your research. If you are not sure, read the appropriate sections before making your selection.

☐ Life sciences ☐ Behavioural & social sciences ☒ Ecological, evolutionary & environmental sciences

For a reference copy of the document with all sections, see nature.com/documents/nr-reporting-summary-flat.pdf

Ecological, evolutionary & environmental sciences study design

All studies must disclose on these points even when the disclosure is negative.

Study description	In this study we expand the genomic information and produce the first reference genomes for heritable (symbiotic) Rickettsia and its sister lineage Candidatus Megaira. We performed a large-scale comparative genomic analysis to clarify the evolution of these neglected groups.
Research sample	We first used a targeted sequencing approach to produce genome assemblies for the Rickettsia symbiont from midge (Culicoides impunctatus), bed bug (Cimex lectularius), tsetse fly (Glossina morsitans submorsitans), and a spider mite (Bryobia graminum) hosts. Additionally, we sequenced and constructed draft genomes for Ca. Megaira from the alga (Carteria cerasiformis). We further extracted assembled 22 genomes (21 Rickettsia and 1 Ca. Megaira) from publicly available arthropod genome sequencing projects (SRA-NCBI). All invertabrate SRA in ncbi were examined, the resulting pool of samples included beetles, bugs, wasps, stoneflies (all listed in table 2. It is reflective of the samples of previously sequenced insects. Many of these do not have metadata for gender or age range. There was no manipulation involved
Sampling strategy	We have previously identified SRA deposits from all available arthropod WGS studies in ncbi containing Rickettsia sequences (https://doi.org/10.1093/gigascience/giab021). All identified deposits were used for subsequent genome mining. non-ncbi genomes were targeted based on the fact that they were infected with rickettsia or megaira. Sample sizes were sufficient because they included all information available to us. We did not aim to compared frequency of infection with host sample size due to the inherent biases within SRA sequences toward laboratory and model organisms. Our aim was to scrape asmuch information about symbiotic bacteria from existing data as possible. For the targeted samples, they were organisms that we knew had symbionts.
Data collection	Raw reads from Rickettsia-containing SRA deposits were downloaded through the European Nucleotide Archive (ENA). Previously published Rickettsia genomes where obtained from GenBank or ENA database. Excel spreadsheets, csv files, tsv files and database files (anvio 7) collected and recorded information during following bioinformatic processes.
Timing and spatial scale	Targeted sequencing efforts took place between 2016-2020. The SRA-NCBI read deposits were downloaded and processed between 2019-2020. This was an almost entirely random screening effort aimed at picking out symbiont genomes dissociated from spatial and temporal metadata. Ecological data is unavailable or irrelevant for 90% of our samples
Data exclusions	All genome assemblies generated in this study and genomes retrieved from the database were quality checked and the ones that did not pass our criteria (completeness > 90% and contamination < 2%) were excluded from downstream analyses.
Reproducibility	The data sources and methods used here are entirely reproducible. All genomes can be downloaded and binned with the same algorithms to produce the same results.<br>The exact methods, tools and code used to obtain and assemble the Rickettsia/Megaira genomes from the SRA deposits and the scripts for downstream comparative analyses are available in github repository (https://github.com/VibrantStarling/Code-used-to-extract-bacterial-genomes-from-invertebrate-genomes) and in supplementary data. All genome metadata and source information can be found in the supplementary data.
Randomization	Largely not relevant as there was no manipulation of sample groups. Our sample collection was only as random as the ncbi SRA database is for arthropods. SRA deposits were grouped by host taxonomy on the ncbi servers. The obtained genomes were assigned to known groups based on phylogeny, ANI/AAI scores, and previous grouping conventions for RIckettsia and Megaira.
Blinding	Is not relevant to this study because there is no manipulation of the original samples and the bacterial genomes extracted were completely unknown to us anyway.

Did the study involve field work? ☒ Yes ☐ No

Field work, collection and transport

Field conditions	Culicoides impunctatus samples were collected from a wild population in Kinlochleven, Scotland on the evenings of the 2nd and 3rd September 2020. Weather was cloudy and still and ~14 degrees Celsius.
Location	Kinlochleven, Scotland (56° 42' 50.7''N 4° 57' 34.9''W), 305m altitude. All other specimen used are either labaoratory strains or are from previous studies.
Access & import/export	Culicoides impunctatus (the highland midge) is very prevalent in Scotland and no particular permit is needed for collections. The

	collected sample was rather small for the population size. Carteria was purchased through the NIES algal culture collection from Japan. All other specimen used were either already sequenced or are from previous studies.
Disturbance	No habitat disturbance was caused. Insects were collected by aspiration. This was the only organism collected by our group for this study. All other samples were laboratoory strains or from previous studies.

Reporting for specific materials, systems and methods

We require information from authors about some types of materials, experimental systems and methods used in many studies. Here, indicate whether each material, system or method listed is relevant to your study. If you are not sure if a list item applies to your research, read the appropriate section before selecting a response.

Materials & experimental systems

n/a	Involved in the study
☒	☐ Antibodies
☒	☐ Eukaryotic cell lines
☒	☐ Palaeontology and archaeology
☐	☒ Animals and other organisms
☒	☐ Human research participants
☒	☐ Clinical data
☒	☐ Dual use research of concern

Methods

n/a	Involved in the study
☒	☐ ChIP-seq
☒	☐ Flow cytometry
☒	☐ MRI-based neuroimaging

Animals and other organisms

Policy information about studies involving animals; ARRIVE guidelines recommended for reporting animal research

Laboratory animals	Strain and culture information for bedbug ('S1' isofemale colony maintained at the University of Bayreuth"), these were kindly provided by Pangupong Thongprem and Oliver Otti and not maintained by the authors. Bedbug culture conditions are described here https://doi.org/10.3389/fmicb.2020.608763 . Carteria cerasiformis strain NIES 425 was obtained from the Microbial Culture Collection at the National Institute for Environmental Studies. A Bryobia mite community was sampled from herbaceous vegetation in Turku, Finland. The Moomin isofemale line was established by isolating a single adult female and was maintained on detached leaves of Phaseolus vulgaris L. cv Speedy at 25 °C, 60 % RH, and a 16:8 light:dark photoperiod. The Moomin spider mite line was morphologically identified as Bryobia graminum by Prof Eddie A. Ueckermann (North-West University).
Wild animals	Culicoides impunctatus females were collected from a wild population in Kinlochleven, Scotland (56° 42' 50.7"N 4° 57' 34.9"W) on the evenings of the 2nd and 3rd September 2020 by aspiration. The Glossinia morsitans submorsitans specimen Gms8 was collected in Burkina Faso in 2010. Both were killed in 100% ethanol and stored at -80 degrees celcius for the purpose of DNA extraction.
Field-collected samples	not applicable
Ethics oversight	Organisms in this study are not covered by ethical legislation

Note that full information on the approval of the study protocol must also be provided in the manuscript.
