# Supplementary material for: Genomic diversity across the Rickettsia and ‘Candidatus Megaira’ genera and proposal of genus status for the Torix group
Source: Nat Commun. 2022 May 12;13:2630. doi: 10.1038/s41467-022-30385-6 (PMC9098888; doi:10.1038/s41467-022-30385-6)
Supplement: Supplementary file 4 — Description of Additional Supplementary Files [file 41467_2022_30385_MOESM4_ESM.pdf]

Title: Supplementary Data 1.
Description: Metadata for all genomes used in this study, alongside their gene content, metabolic and phylogenomic data.
